# Advanced Endoscopic Rescue of a Complication (Duodenojejunostomy Leak) After a Pylorus-Preserving Pancreaticoduodenectomy in a Post-Esophagectomy Patient with Pancreatic Adenocarcinoma: A Case Report and Review of the Literature

**DOI:** 10.1089/pancan.2019.0016

**Published:** 2020-02-06

**Authors:** Stephanie E. Honig, Megan P. Lundgren, Thomas E. Kowalski, Harish Lavu, Charles J. Yeo

**Affiliations:** Department of Surgery, The Jefferson Pancreas, Biliary and Related Cancer Center, Sidney Kimmel Medical College, Thomas Jefferson University, Philadelphia, Pennsylvania.

**Keywords:** duodenojejunostomy leak, endoscopy, esophagectomy, pancreatic adenocarcinoma, pylorus-preserving pancreaticoduodenectomy

## Abstract

**Background:** Approximately 4% of patients develop a second upper gastrointestinal cancer after esophagectomy, and nearly 60,000 people are diagnosed with pancreatic cancer in the United States each year. The need for a Whipple procedure after esophagectomy is rarely reported. Post-esophagectomy anatomy, particularly the vascular supply, makes this a complex operation. Herein, we describe the advanced endoscopic rescue of a duodenojejunostomy (DJ) leak after pylorus-preserving pancreaticoduodenectomy (PPPD) in a post-esophagectomy patient.

**Presentation:** A 72-year-old male with a remote history of esophageal cancer treated with minimally invasive three-hole esophagectomy and chemoradiation presented to our institution for evaluation and management of newly diagnosed pancreatic cancer. The patient had undergone common bile duct (CBD) stent placement by his gastroenterologist 2 weeks earlier after experiencing jaundice, weight loss, and steatorrhea. Endoscopic ultrasound confirmed the presence of a pancreatic head and neck mass, obstructing and dilating the main pancreatic duct and CBD. Fine-needle biopsy revealed a poorly differentiated adenocarcinoma. A PPPD was performed without intraoperative complications. The patient was subsequently readmitted with a DJ leak requiring interventional radiology and advanced endoscopic intervention.

**Conclusions:** PPPD in patients with pancreatic cancer can be performed after previous esophagectomy. Careful dissection is crucial to avoid injury to the remaining right gastric and right gastroepiploic arteries that supply the gastric conduit after esophagectomy. The DJ is at risk after this operation, and access to tertiary care inclusive of interventional radiology and advanced endoscopic teams is critical to the correction and healing of a leak of this anastomosis.

## Introduction

Nearly 60,000 people are diagnosed with pancreatic cancer in the United States each year.^1^ Patients with pancreatic cancers that occur in the head or neck of the pancreas and are without vascular involvement can be surgically treated by pancreaticoduodenectomy (Whipple procedure), utilizing either a classic or pylorus-preserving technique, combined with neoadjuvant or adjuvant chemotherapy and radiation.^2,3^ With increasingly safe and effective surgical options at high-volume centers as well as efficacious neoadjuvant and adjuvant therapies, more patients will survive upper gastrointestinal (UGI) cancers and over time develop second primary cancers. Approximately 4% of patients develop a second UGI cancer after esophagectomy, and there are a few cases of operative management of head of pancreas cancers in post-esophagectomy patients.^4–7^ These cases require complex preoperative planning and intraoperative decision making related to altered anatomy and vascular supply, as well as a heightened readiness for postoperative complications. In this article, we present a case of pancreatic adenocarcinoma managed by pylorus-preserving pancreaticoduodenectomy (PPPD) after previous esophagectomy for esophageal cancer, complicated by a duodenojejunostomy (DJ) leak. The management of this leak is outlined.

## Case Presentation

A 72-year-old physically active retired family physician presented to our institution for evaluation and management of newly diagnosed pancreatic cancer. The patient was seen by his gastroenterologist 2 weeks before presentation with symptoms of jaundice, weight loss, and steatorrhea, and he underwent common bile duct (CBD) stent placement for obstructive jaundice at that time. Past medical history is notable for esophageal cancer treated with three-hole minimally invasive esophagectomy in 2008. Pathology at that time revealed moderately differentiated adenocarcinoma involving both esophageal and gastric mucosa, with 12 out of 21 examined lymph nodes positive for tumor. All surgical margins were negative for residual tumor. The patient subsequently underwent chemoradiation due to lymph node involvement. The surgical history also included a post-esophagectomy laparoscopic diaphragmatic hernia repair in 2011. Notably, the patient had smoked cigars for the past 15 years.

**FIG. 1.**
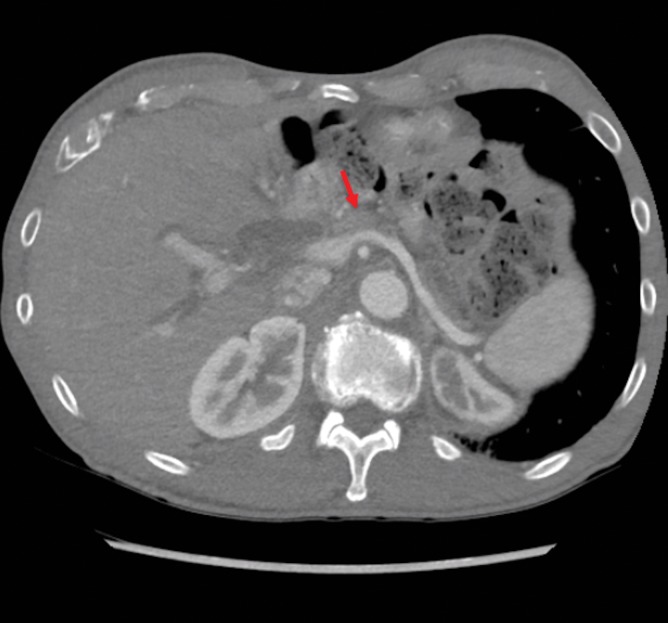
CT image revealing a pancreatic neck mass (arrow) measuring 2.2 × 1.3 cm, with dilated intrahepatic bile ducts and patent splenic vein and portal vein. CT, computed tomography.

**FIG. 2.**
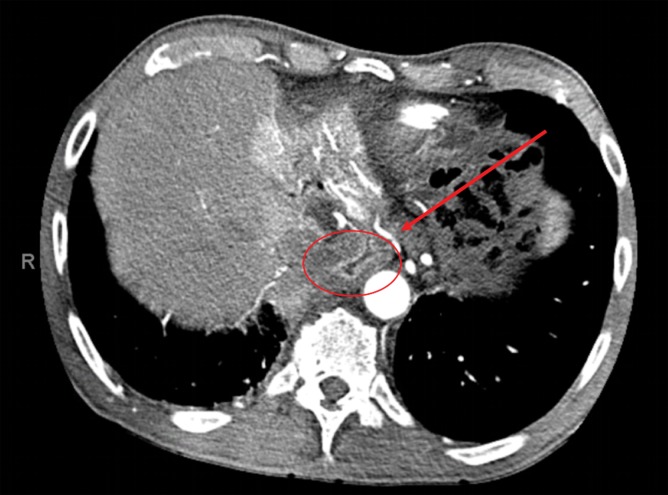
Preoperative CT angiography illustrating the common hepatic artery dividing into the proper hepatic artery, gastroduodenal artery, and right gastric artery (arrow) that is supplying the gastric conduit (circle).

On presentation to our surgical clinic, scleral icterus and mild jaundice were noted. Laboratory studies revealed hyperbilirubinemia and elevated liver function tests. Both CA 19-9 and carcinoembryonic antigen levels were within normal range. Computed tomography (CT) scan revealed a pancreatic neck mass measuring 2.2 × 1.3 cm with obstruction of the CBD and main pancreatic duct (MPD) as well as possible superior mesenteric vein (SMV) involvement. Significant gallbladder distention secondary to biliary obstruction was also noted (Fig. 1). Endoscopic ultrasound confirmed the presence of a pancreatic head and neck mass obstructing and causing dilation of the MPD and CBD. Fine-needle biopsy of the mass revealed a poorly differentiated adenocarcinoma. Given the patient's prior history of esophagectomy, a CT angiogram was performed and confirmed blood supply to the gastric conduit via the right gastric artery, right gastroepiploic artery, and gastroduodenal artery (GDA; Fig. 2). Knowing that the right gastric artery was patent, a PPPD was planned to resect the pancreatic neck mass with the intent to sacrifice the GDA. The patient refused preoperative chemotherapy.

**FIG. 3.**
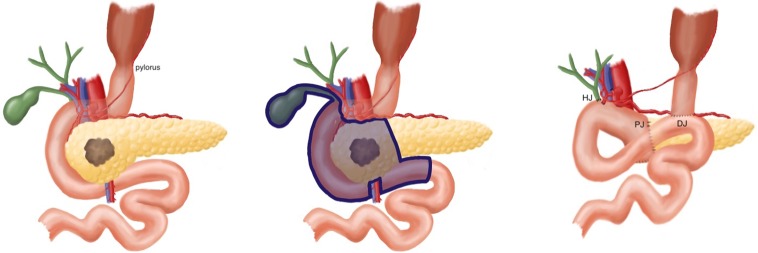
Diagram of the pylorus-preserving pancreaticoduodenectomy procedure (illustration by Stephanie E. Honig).

At the time of surgery, no dissemination was noted. A majority of the stomach was in the chest and not seen in the operative field. The pylorus was identified just below the diaphragm, as well as the first and second parts of the duodenum. Dissection of the duodenum off of the anterior aspect of the pancreatic head and neck was difficult because of the altered anatomy and the gastric pullup. The duodenum was carefully divided at least 3 cm beyond the pylorus, ensuring that the right gastric and right gastroepiploic arteries were left intact. The GDA was identified, suture ligated, and divided at its takeoff from the common hepatic artery. The proximal jejunum was divided about 20 cm below the ligament of Treitz. The duodenojejunal junction was mobilized, and the specimen was carefully separated from the adjacent mesenteric vessels. The pancreatic neck was divided to the left of SMV-portal vein confluence. The pancreatic head and neck were meticulously dissected from the right lateral aspect of the SMV and portal vein, and the specimen was separated from the visceral vessels. Typical end-to-side pancreatojejunostomy (PJ) and end-to-side hepaticojejunostomy were created. An end-to-side DJ was created in the normal fashion. However, due to the patient's previous esophagectomy, the duodenal stump was located very high in the abdomen, just inferior to the crus of the diaphragm, making the DJ anastomosis technically difficult (Fig. 3). A right-sided drain and a left-sided drain were placed; the left drain passed between the PJ and the DJ, which, due to post-esophagectomy anatomy, were in close proximity.

Final pathology of the resected pancreatic mass confirmed poorly differentiated adenocarcinoma with perineural invasion and invasion into peripancreatic adipose tissue (Fig. 3). Genetic analysis was positive for KRAS mutations in codons 12, 13, 61, 117, and 146 of known pathologic significance in pancreatic adenocarcinoma. The pancreatic neck margin on the specimen was positive for adenocarcinoma, but no gross tumor was left in place. The patient recovered uneventfully and per the Whipple accelerated recovery pathway (WARP protocol)^9^ was discharged home on postoperative day 6 on a full liquid diet with his left-sided drain still in place as its fluid was high in triglycerides, suggesting a chyle leak.

**FIG. 4.**
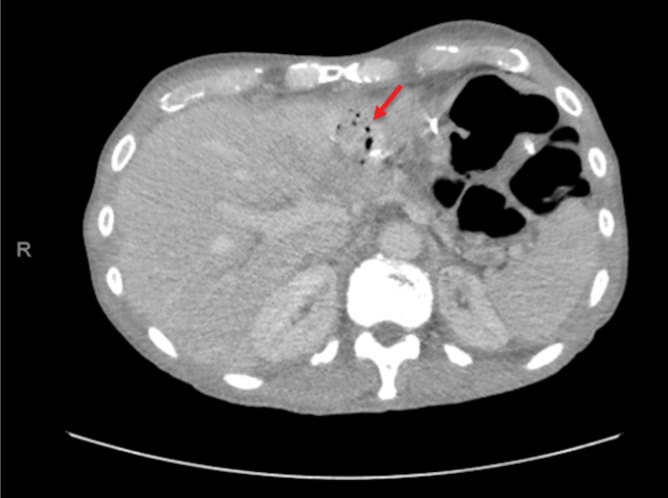
CT scan showing 7-cm collection with fluid and air (arrow) adjacent to the DJ and PJ. DJ, duodenojejunostomy; PJ, pancreatojejunostomy.

**FIG. 5.**
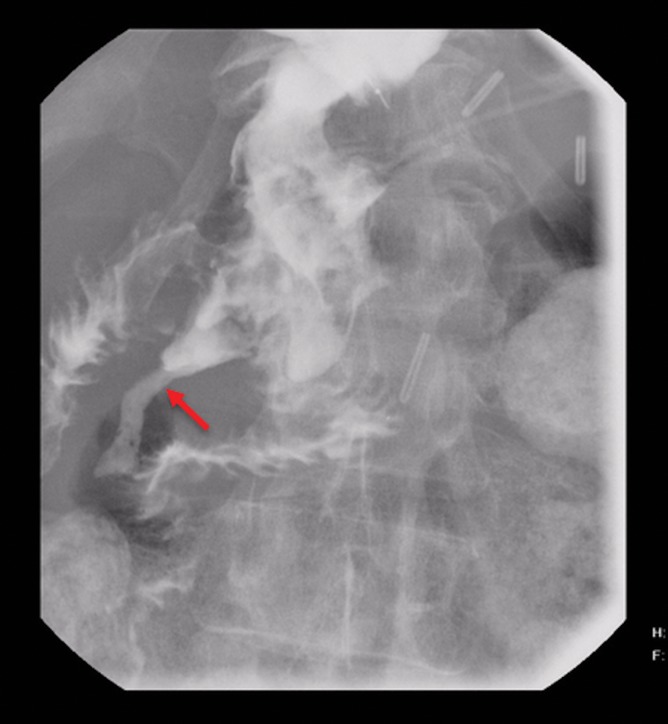
UGI series showing DJ leak (arrow). UGI, upper gastrointestinal.

The patient was seen in the outpatient office at 2 weeks post-discharge. The drain output was discolored and the patient appeared dehydrated, so he was sent for a CT scan (Fig. 4). This revealed a 7-cm collection with fluid and air near the DJ and PJ. Antibiotics were started. A peripherally inserted central catheter was placed, and total parenteral nutrition (TPN) was commenced. Interventional radiology injected the existing surgical drain with contrast, which opacified the duodenum and gastric pullup, and the drain location was optimized. An UGI swallow revealed extravasation from the DJ, suggestive of a leak. A nasogastric tube was placed into the gastric pullup. A normal gastric pH was achieved by using proton pump inhibitors. Drain output decreased significantly over the course of 3 days. A repeat UGI was performed and revealed a continued leak, with patent efferent and afferent limbs of the anastomosis (Fig. 5). A CT scan revealed a collection remaining posterior to the xyphoid process. The surgical drain was interrogated and left in place. A pigtail drain was placed into the retro-xyphoid collection by interventional radiology.

At this time, the Division of Gastroenterology's Pancreaticobiliary and Advanced Endoscopy section was consulted for endoscopic management. Considerations at that time were primary endoscopic closure, vacuum-assisted closure, or enteral stent placement. The procedure was performed under fluoroscopy. Endoscopy revealed a healthy appearing gastric pullup with patent esophagogastric anastomosis. The defect at the DJ anastomosis was large, and the percutaneously placed pigtail catheter was seen partially within the jejunal lumen. The choice of endoscopic closure was based on anatomic and technical considerations. To this end, the defect was large, at an acute angle to the lumen/axis of the endoscope, and in a small space within which it was difficult to operate. As such, the defect was too large for closure with an over the scope clip (OVESCO) and the space/orientation would not allow for closure by using the endoscopic suturing device (Overstitch, Apollo). Endoscopic vacuum-assisted closure (EVAC) has primarily been described for anastomotic defects closer to the mouth (esophageal) or the anus (rectal), as it requires endoscopically advancing a polyurethane sponge tied to a nasogastric tube into the defect. The sponge must be changed/downsized every 2 to 5 days while the patient remains in the hospital. The duodenal location and difficult access to the DJ anastomosis in this case favored the use of fully covered stents for attempted closure.

**FIG. 6.**
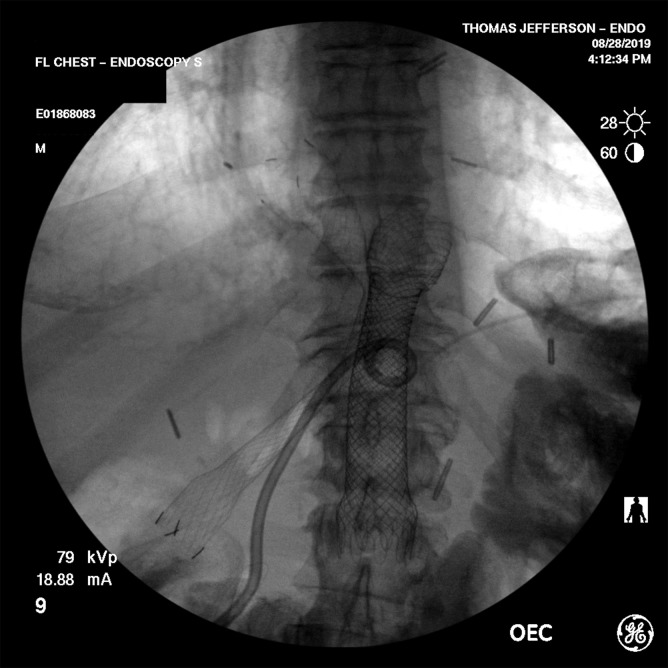
A FCSEMS placed across the DJ into the efferent limb, and a second FCSEMS placed into the afferent limb. The percutaneous interventional radiology drain is also seen. FCSEMS, fully covered self-expanding metal stent.

A pediatric scope was utilized to navigate the afferent limb, after which a guidewire was passed through the scope and retained. The efferent limb was notably more difficult to navigate but ultimately access was achieved, and a guidewire was advanced for stent placement. A therapeutic upper endoscope was passed over the efferent limb wire and an 18 mm by 103 mm fully covered self-expanding metal stent (FCSEMS; Niti-S, TaeWoong) was placed across the anastomosis into the efferent limb. Similarly, an 18 mm by 100 mm FCSEMS was placed into the afferent limb (Fig. 6). Due to the significant risk of migration of fully covered metal stents, we secured the proximal aspect of the stents to the gastric wall with three sutures by using the endoscopic suturing device (Overstitch, Apollo).

**FIG. 7.**
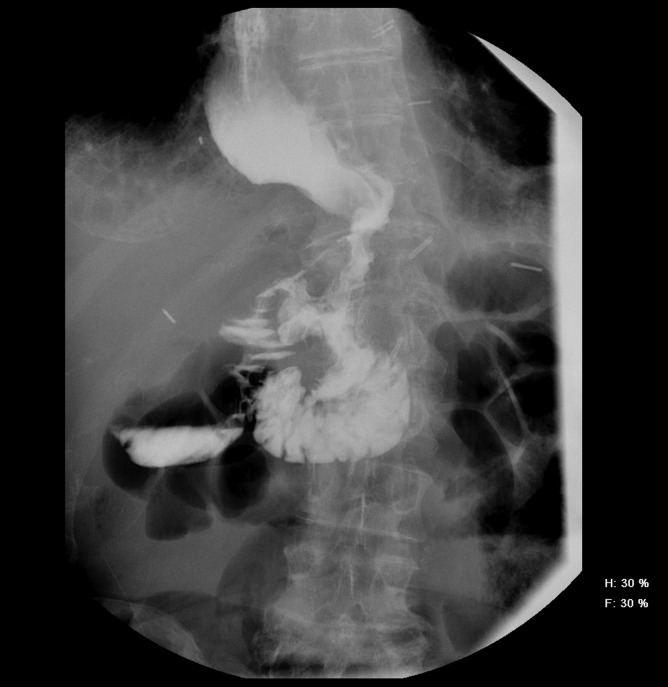
Post-stent removal UGI series showing no leak or obstruction.

The patient was discharged home a few days after stent placement on continuous TPN with drain care. During his follow-up visit 2 weeks after discharge, his drains had scant output and were removed. He visited a medical oncologist, with plans to discuss adjuvant therapy options for the near future. At the last surgical follow-up on postoperative day 55, he appeared well nourished and his recovery was progressing smoothly. An UGI series with the stents in place revealed no extravasation and good stent location. His TPN was discontinued after 6 weeks, and he has been recommenced on oral alimentation. His stents were successfully removed endoscopically on postoperative day 77, and a post-stent removal UGI series appeared normal with no leaks (Fig. 7).

## Discussion

Pancreatic cancer occurs in nearly 60,000 patients per year in the United States, with adenocarcinoma comprising 85% of these tumors. Pancreatic cancer is the third leading cause of cancer-related deaths in both men and women in the United States.^1^ Our patient was diagnosed with pancreatic adenocarcinoma 11 years after treatment for esophageal cancer with chemoradiation and esophagectomy. There are no reported cancer syndromes or genetic mutations known to link pancreatic and esophageal cancers. However, there is a strong association with tobacco use in both pancreatic and esophageal cancers. The risk of pancreatic cancer is 1.5 times higher in smokers as compared with non-smokers.^10^ In addition, cigar smoking, in particular, doubles the risk of UGI tract cancers.^11^

Pancreatic malignancies that are localized to the head or neck of the pancreas are typically treated surgically with a pancreaticoduodenectomy (Whipple procedure), utilizing either the classic or pylorus-preserving technique. There are a few case reports of pancreaticoduodenectomy performed on patients with a history of esophagectomy, all of which describe different approaches to these complex cases (Table 1). Our patient underwent a PPPD due to his unusual gastrointestinal anatomy with the antrum of the stomach sitting in the chest, making a classic Whipple procedure difficult. We performed a PPPD without preservation of the GDA since preoperative imaging demonstrated a patent right gastric artery.

**Table 1.** Previous Cases Describing Different Approaches to Pancreaticoduodenectomy Performed on Patients with a History of Esophagectomy

Study	Case	Surgical approach
Fragulidis et al.^5^	A 50-year-old male with history of esophagectomy for esophageal cancer 13 years before presentation	Preservation of GDA and gastroepiploic arteries
Addeo et al.^6^	A 73-year-old male with history of right nephrectomy and lower esophagectomy 6 years before presentation	Careful dissection avoiding injury to gastroepiploic vessels
Kim et al.^7^	A 65-year-old male with concomitant esophageal and pancreatic cancers	Combined esophagectomy and PPPD using “supercharged” jejunal conduit constructed to replace resected esophagus
Ikeda et al.^8^	1. A 61-year-old male with history of proximal gastrectomy with anastomosis between esophagus and remnant distal stomach for gastric carcinoma 10 years before presentation	1. Careful identification and preservation of the right gastroepiploic vessels
	2. A 63-year-old male with history of subtotal esophagectomy for esophageal cancer 10 years before presentation	2. Preservation of GDA and right gastroepiploic artery

GDA, gastroduodenal artery; PPPD, pylorus-preserving pancreaticoduodenectomy.

The option of a conduit was not entertained intraoperatively in the context of requiring a total gastrectomy with colonic or jejunal conduit, which would have required access to the thoracic cavity. At the time of the operation, tension at the anastomosis was not of major concern, despite the anastomosis being at the level of the diaphragm. It did not appear to be under tension at the conclusion of the case. The primary preoperative concern was blood supply to the pylorus, and we were relying on the right gastric artery that was patent on preoperative CT angiogram images. A conduit would have mitigated blood supply concerns that were discussed preoperatively. However, this would have required a more extensive operation in two body cavities.

The patient's course was complicated by a DJ leak classified as a Clavien grade IIIb complication, as it was managed with endoscopic intervention requiring general anesthesia.^12^ Provided that there was a pre-existing percutaneous drain in the region of the defect, it would always be our preference to primarily close the defect with either an over the scope clip or endoscopic suturing. Unfortunately, due to anatomic and technical constraints, it was felt that neither of these modalities would be successful.

EVAC has been infrequently described for duodenal/pancreaticobiliary defects with good outcomes. The most significant risk of EVAC is significant hemorrhage from vessels contiguous with the defect. Based on the vasculature in the region of the DJ anastomosis, the bleeding risk seems to be higher than has been reported in the literature. In addition, the need to change the sponge every 2 to 5 days is arduous and places the patient at risk of repeated endoscopic procedures.

The patient has recovered quite well after advanced endoscopic exclusion of the defect from the gastrointestinal lumen without afferent limb occlusion via placement of two fully covered metal stents. The primary risk of fully covered metal stents is failure to seal the defect. Fully covered metal stents will help divert luminal contents away from the defect but often do not result in a fluid/air competent closure. As always, competency of closure needs to be assessed by using luminal contrast studies and monitoring percutaneous drain output. Migration is another risk of fully covered metal stents. Migration can result in stents becoming lodged in the small intestine requiring deep enteroscopy or surgery to remove. For this reason, all fully covered metal stents are generally secured to the gastrointestinal wall by using clips or sutures.

## Conclusions

A PPPD is possible after esophagectomy, taking care to preserve the blood supply to the gastric conduit and proximal duodenum. Complex pancreas surgery, particularly in patients with altered UGI anatomy, is best performed at a high-volume center with the availability of expert interventional radiology and advanced endoscopic capabilities.

## Abbreviations Used

CBD: common bile duct
CT: computed tomography
DJ: duodenojejunostomy
EVAC: endoscopic vacuum-assisted closure
FCSEMS: fully covered self-expanding metal stent
GDA: gastroduodenal artery
MPD: main pancreatic duct
PJ: pancreatojejunostomy
PPPD: pylorus-preserving pancreaticoduodenectomy
SMV: superior mesenteric vein
UGI: upper gastrointestinal
